# Effects of taichi on grade 1 hypertension: A study protocol for a randomized controlled trial

**DOI:** 10.1186/s13063-019-4028-6

**Published:** 2020-02-13

**Authors:** Sang-Hyun Lee, Byung-Jun Kim, In-Hwa Park, Eui-Hyoung Hwang, Eun Ju Park, Insoo Jang, Man-Suk Hwang

**Affiliations:** 1 Department of Rehabilitation Medicine of Korean Medicine, Spine and Joint Center, Pusan National University Korean Medicine Hospital, 20, Geumo-ro, Mulgeum-eup, Yangsan-si, Gyeongnam 50612 Republic of Korea; 2 School of Korean Medicine, Pusan National University, Yangsan, Republic of Korea; 3 Division of Clinical Medicine, School of Korean Medicine, Pusan National University, Yangsan, Republic of Korea; 4 Family Medicine Clinic, Pusan National University Yangsan Hospital, Yangsan, Republic of Korea; 5 Department of Internal Medicine, College of Korean Medicine, Woosuk University, 443, Samnye-ro, Samynye-eup, Wanju-Gun, Jeonbuk Republic of Korea

**Keywords:** Hypertension, Taichi, Taichichuan, Taijiquan, Martial arts, Blood pressure

## Abstract

**Background:**

Medication is generally recommended to reduce the morbidity and mortality caused by cardiovascular disease in hypertensive patients. However, considering the difficulties and economic factors associated with long-term medication, interest in taichi as an exercise treatment method has increased recently in Korean medical practice. Numerous studies have suggested that taichi can be used to treat various diseases and that is can affect psychosomatic factors such as anxiety. This study aims to evaluate the effect of taichi in reducing blood pressure among grade 1 hypertensive patients.

**Methods/design:**

In this randomized, active-controlled, assessor-blinded, two parallel-armed trial, 80 grade 1 hypertension patients will be recruited and randomly assigned to the usual care group or to the taichi group (*n* = 40 in each group). Subjects who voluntarily sign a study agreement will be educated in managing their own blood pressure by restricting salt intake, losing weight, moderating alcohol consumption, performing exercise, and regulating dietary intake at their first visit. In addition to self-management, the taichi group will perform two 60-min taichi sessions per week for a total of 8 weeks. Blood pressure will be measured as the primary outcome. In addition, body composition, heart rate, and the perceived intensity and difficulty of the exercise will be measured as secondary outcomes.

**Discussion:**

This study is a randomized controlled trial of taichi, which is not widely practiced in Korea. It may provide valuable data on the effects of taichi on hypertension, which will inform non-pharmaceutical treatment options for this disorder.

**Trial registration:**

Clinical Research Information Service, KCT0003632. Registered on 18 March 2019.

## Background

According to the Korea National Health and Nutrition Examination Survey (2015), the prevalence of hypertension is 27.9% in Korea, and 36.8% of Korean adults belong to the pre-hypertension category [1]. The prevalence of age-related hypertension was 52.1% in males and 51.5% in females aged 60–69 years, and 61.7% in males and 71.3% in females aged 70 years or older [1]. The higher prevalence in older patients is because age-related reductions in blood vessel elasticity increase the systolic blood pressure (SBP) and decrease the diastolic blood pressure (DBP) [2]. If blood pressure is not controlled, blood vessel damage will occur and lead to the complications of hypertension [2]. If hypertension is not detected early and treated properly, it can lead to complications such as myocardial infarction, stroke, and kidney failure [3].

The goal of controlling hypertension is to reduce the morbidity and mortality caused by cardiovascular disease by maintaining normal blood pressure [4, 5]. Medication for hypertension is generally recommended for SBP greater than 140 mmHg in patients younger than 60 years of age or DBP greater than 90 mmHg regardless of age [6]. However, considering the side effects and economic factors associated with long-term medication, non-medication approaches, such as eating healthily, exercising, ceasing smoking, and moderating alcohol consumption, are widely used to treat and manage hypertension along with pharmacotherapy [7]. In particular, Parker et al. [8] reported that physical activity, including moderate-intensity exercises such as walking or home exercise, reduced the blood pressure in incident hypertension after 15 years of follow-up.

In recent years, there has been increasing interest in taichi as an exercise treatment method for various diseases [9–15]. According to a study conducted by Kim [16], taichi may lower sympathetic tone and increase parasympathetic tone, which may result in changes in the autonomic nervous system. In addition, a randomized controlled study conducted by Tsai et al. [17] suggested that a 12-week period of taichi exercise reduces blood pressure, as well as lipid levels, and improves patient anxiety.

In this study, we analyze the blood pressure, heart rate, and body composition of grade 1 hypertension patients who have been diagnosed during a medical examination in a hospital or had an SBP of 140–159 mmHg or DBP of 90–99 mmHg in a screening test. We will evaluate the effectiveness of taichi by comparing the control of hypertension in a group practicing usual care with that in an experimental group practicing usual care plus taichi. The effectiveness of taichi for hypertension will, thus, be assessed in this randomized, active-controlled, assessor-blinded, two parallel-armed trial.

## Methods/design

### Study design

The proposed study is a randomized, active-controlled, assessor-blinded, two parallel-armed trial. It will be conducted at Pusan National University Korean Medicine Hospital (PNUKH) in Yangsan, Korea. The study protocol was approved by the institutional review board (IRB) of PNUKH on 16 January 2019 (approval number 2018014) and was registered in the Clinical Research Information Service on 18 March 2019 (KCT0003632). Table 1 is the schedule of enrollment, intervention, and assessments. Table 2 lists all relevant items in the World Health Organization’s trial registration data set.

**Table 1:** Schedule of enrollment, intervention, and assessments

Measure	Screening (week 0)	Active treatment								Follow-up (12 weeks after screening)^†^
		Week 1	Week 2	Week 3	Week 4 (visit 1)^†^	Week 5	Week 6	Week 7	Week 8 (visit 2)^†^	
Study agreement	X									
Check for participation in other clinical trials	X									
Sociodemographic characteristics^1^	X									
Taichi exercises		Two sessions per week								
Body composition test	X								X	X
Measurement of vital signs^2^	X				X				X	X
Intensity and difficulty of exercises									X	
Number of taichi sessions attended										X
Adverse events					X				X	X

† ±3 days

^1^Age, gender, occupation, past history, present illness, and medications

^2^Blood pressure (systolic and diastolic), heart rate, and body temperature

**Table 2:** All relevant items in the World Health Organization’s trial registration data set

Data category	Information
Primary registry and trial identifying number	Clinical Research Information Service, KCT0003632
Date of registration in primary registry	18 March 2019
Secondary identifying numbers	Not applicable
Source(s) of monetary or material support	Traditional Korea Medicine R&D program of Korea Health Industry Development Institute
Primary sponsor	Woosuk University
Secondary sponsor(s)	Not applicable
Contact for public queries	Man-Suk Hwang, + 82–55–360-5970, hwangmansuk@pusan.ac.kr
Contact for scientific queries	Man-Suk Hwang, Department of Rehabilitation Medicine of Korean Medicine, Spine and Joint Center, Pusan National University Korean Medicine Hospital, 20, Geumo-ro, Mulgeum-eup, Yangsan-si, Gyeongnam 50612, Republic of Korea.
Public title	Effects of taichi on grade 1 hypertension: A study protocol for a randomized controlled trial
Scientific title	The Effects of Taichi on Grade 1 Hypertension: Randomized controlled trial
Countries of recruitment	Republic of Korea
Health condition(s) or problem(s) studied	Hypertension
Intervention(s)	Taichi
Key inclusion and exclusion criteria	Inclusion Criteria o Patients must have been diagnosed with grade 1 hypertension during a health checkup or at the hospital, have SBP of 140 to 159 mmHg, or have DBP of 90 to 99 mmHg. o Patients who understand the study procedures and are able to follow the advice given o Patients must sign the study agreement and voluntarily agree to participate in the study Exclusion Criteria o Patients who have participated in another trial within a month before this study. o Patients whose high blood pressure is deemed by a doctor as too difficult to treat with exercise because of conditions such as severe pain or joint deformation. o Patients who are unable to communicate properly, for example due to dementia or mild cognitive impairment. o Patients who are pregnant. o Patients who should not be included in this study based on the investigator’s judgment.
Study type	Type of Study: Interventional Method of allocation: Randomized Masking: Outcome assessor blinding Assignment: Two-parallel armed, active controlled
Date of first enrollment	17 April 2019
Target sample size	1. Number of patients that the trial plans to enroll in total: 80 2. Number of patients that the trial has enrolled: 19
Recruitment status	Recruiting
Primary outcome(s)	Blood pressure, which will be measured at baseline, prior to each hospital visit, and during the follow-up visit
Key secondary outcomes	Body composition, which will be measured at baseline, week 8, and at the follow-up visit Heart rate, which will be measured at baseline, prior to each visit, and during the follow-up visit Intensity and difficulty of the exercises, which will be assessed once, at the end of the exercise training.
Ethics Review	1. Status: Approved 2. Date of approval: 16 January 2019 3. Name and contact details of Ethics committee: Pusan National University Korean Medicine Hospital IRB (approval number 2018014), 20, Geumo-ro, Mulgeum-eup, Yangsan-si, Gyeongnam 50612, Republic of Korea, + 82–55–360-5902
Completion date	15 January 2020
Summary results	Not applicable: protocol
Individual patient data sharing statement	1. Plan to share individual patient data: Decided 2. Plan description: The datasets used or analyzed during the study can be requested from the corresponding author

### Participants

#### Inclusion criteria

To be included, patients must meet all the inclusion criteria:

- ◦ They must have been diagnosed with grade 1 hypertension during a health checkup or at the hospital, have SBP of 140 to 159 mmHg, or have DBP of 90 to 99 mmHg.
- ◦ They must understand the study procedures and should be able to follow the advice given
- ◦ They must sign the study agreement and voluntarily agree to participate in the study

#### Exclusion criteria

People who meet any of the exclusion cannot participate in the trial, that is

- ◦ If they have participated in another trial within a month before this study.
- ◦ If their high blood pressure is deemed by a doctor as too difficult to treat with exercise because of conditions such as severe pain or joint deformation.
- ◦ If they are unable to communicate properly, for example due to dementia or mild cognitive impairment.
- ◦ If they are pregnant.
- ◦ If they should not be included in this study based on the investigator’s judgment.

#### Discontinuation and dropout criteria

Participants will be withdrawn from the study:

- ◦ If they are found to have a previously undiagnosed severe disease after enrollment and before the start of the clinical trial.
- ◦ If they have another disease (except hypertension) that may influence the results of the trial.
- ◦ If they miss two consecutive assessment visits.
- ◦ If they ask to be withdrawn from the trial.
- ◦ If they are lost to follow-up.

### Recruitment

Patients will be recruited using advertisements on the bulletin boards of Pusan National University Yangsan Hospital (PNUYH) and PNUKH, at local public health centers, and at other local government offices. Patients who are interested in participating will first be screened to determine their eligibility. Eligible patients will receive an explanation of the study and can voluntarily decide whether they wish to participate. If the patient consents to the study terms, a clinical research coordinator (CRC) will check whether they are suitable in accordance with the inclusion and exclusion criteria. Then, the patient will be assigned randomly to the taichi group or the usual care group. After allocation, the CRC will schedule their treatment procedure. The first participant was enrolled on 17 April 2019.

### Randomization

**Fig. 1:**
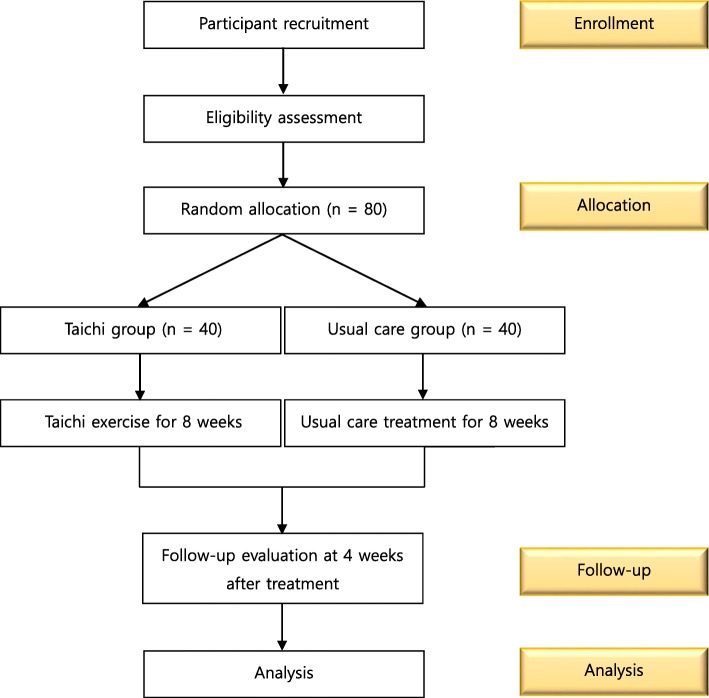
Flowchart outlining the study timeline, including enrollment, allocation, follow-up, and analysis

A statistician who is not involved in conducting or assessing the clinical trial will generate a randomization sequence using the statistical program SAS®, Version 9.4 (SAS institute. Inc., Cary, NC). Using the sequence, the statistician will prepare 80 sealed envelopes, 40 of which will contain an A and 40 a B based on blocked randomization. Once a participant has signed the informed consent form, the CRC will open the next envelope in the sequence and inform the practitioner of the participant’s assignment to the experimental group or the usual care group (Fig. 1).

### Blinding

Since blinding of the intervention is impossible, the group allocation will not be concealed and the participants will not be blind. Instead, the outcome assessor and data analyst will be blind to the allocation and they will not participate in recruitment or the intervention. The outcome assessor will assess participants at the study visits. The data analyst will analyze the statistical data to prevent selective reporting of outcome variables. Unblinding will be permitted only when it is necessary to reveal the participant’s allocated intervention, such as in cases of severe side effects, at the discretion of the assessor.

### Education levels of the practitioners

All the physicians participating in this study as practitioners or researchers are licensed by the Ministry of Health and Welfare of Korea and have at least one year of clinical experience in Korean medicine. These practitioners will be adequately trained so that they closely adhere to the research protocols and are familiar with the research treatment methods. All the taichi educators have been trained as Korean rehabilitation medical trainees or specialists in taichi exercise and have at least one year of taichi experience. Among them, the research director of this study has more than ten years of taichi experience.

### Intervention

At their first visit, patients in both groups will be educated in managing their own blood pressure by restricting salt intake, losing weight, moderating alcohol consumption, exercising, and regulating their diet. Additionally, patients in both groups will be asked not to do any intense exercise that could influence the results of the trial. After this education, the usual care group will self-manage their own blood pressure for 8 weeks.

In addition to self-management, the experimental group will perform taichi as the study intervention. The taichi used in this study is Chen-style 18-form taichi, which will be conducted over two 60-min sessions per week for a total of 8 weeks (80% compliance and more than 13 sessions in total). In each session, patients will perform 10 min of warm-up exercises, 40 min of taichi, and 10 min of cool-down exercises. To maximize adherence to the study protocol, the intervention will mostly be performed in the 6th floor ward at PNUKH. If group sessions are needed, the practitioners can visit external locations. The patients in the experimental group will receive a reference book on taichi that will promote their retention of the exercises. A daily log will record how many exercises each patient has conducted. At weeks 4 and 8, both groups will visit the hospital for an assessment. After treatment, both groups will be monitored for an additional 4 weeks of follow-up.

### Outcome assessment

At the initial screening visit, the CRC will explain the study protocol to the patient. Then, the participants will be asked about their sociodemographic characteristics, including age, gender, occupation, past history, present illness, and medications, at an isolated room for allocation concealment. All adverse events will be recorded, and the practitioners will check the severity of the events and decide the continuance of the trial. Follow-up assessments will be performed once at 12 weeks after the initial screening visit (Table 1, Fig. 1).

#### Primary outcome measurements

Blood pressure will be the primary outcome of this trial. The CRC will assess the participant’s blood pressure in a stable state using an automatic electronic blood pressure monitor (HBP-1300, Omron Dalian Co., Ltd., China). To obtain accurate data, the measurements will be conducted three times and their mean value will be used as the outcome. Blood pressure will be measured at baseline (assessment 1), prior to each of the visits (assessments 2 and 3), and during the follow-up visit (assessment 4). The primary endpoint is week 8 (assessment 3).

#### Secondary outcome measurements

Body composition is one of the secondary outcomes of this trial. The body composition test assesses each participant’s weight, body fat mass, body mass index, percentage of body fat, and weight-to-hip ratio. The participant will stand barefoot on the Inbody body composition analyzer (Inbody 770; Inbody Co., Ltd., South Korea). After their weight has been measured, the participant will grasp the handles and the machine will pass multifrequency signals through their body to obtain the impedance corresponding to each frequency. Using these measured impedance values, the machine will calculate how much body fat they have. The test will be conducted at baseline (assessment 1), week 8 (assessment 3), and at the follow-up visit (assessment 4).

Heart rate is another secondary outcome of this trial. The heart rate of participants will assessed when they are in a stable state at the same time as their blood pressure is measured. Thus, measurements will be conducted at baseline (assessment 1), prior to each of the visits (assessments 2 and 3), and during the follow-up visit (assessment 4).

The intensity and difficulty of the exercises are also secondary outcomes. The aim is to compare their subjective assessment of the intensity of the exercise with its absolute intensity. The experimental group will be asked four questions.

1. Was this exercise routine easy to follow?
2. Is taichi useful for improving your health?
3. Was the taichi conducted at appropriate time for you?
4. Is the reference book helpful for performing taichi on your own?

Participants will answer each question using a 0 to 10 category scale.

They will rate the difficulty of the taichi exercises using a 0 to 10 visual analog scale, on which 0 indicates no difficulty and 10 indicates the maximum possible difficulty the person can imagine. Both surveys will be conducted once at the end of the final taichi session. Thus, the measurement point will be week 8 (assessment 3).

### Sample size

This study took into account the results of a previous study [18] that used taichi as the main evaluation index. The calculated sample size necessary for the *t*-test was 36 subjects in each group, which was conducted with a G power analysis with an effect size of 0.67, test power of 0.80, and significance level of 0.05. Considering a dropout rate of 10%, we aim to recruit a total of 40 subjects in each group.

### Statistical analysis plan

Continuous variables will be expressed as mean ± standard deviation, and categorical variables will be expressed as *n* (%). The demographic baseline information (age, gender, occupation, past history, present illnesses, and medications) will be tested using the chi-squared test and independent *t*-test.

As the primary statistical analysis, the effectiveness of taichi will be tested by calculating the differences in the maximum SBP and minimum DBP before (baseline) and after (week 8) treatment for each test subject. Comparisons of the within-group differences will be performed using a paired *t*-test and between-group differences using an independent *t*-test. If the data do not have a normal distribution, they will be tested using a nonparametric test (a Wilcoxon signed-rank test or Wilcoxon rank-sum test).

The heart rate and the body composition of the participants will be tested to verify the effectiveness of taichi by calculating the difference between before (baseline) and after (week 8) the treatment as described above.

The results of the questions regarding the intensity and difficulty of the exercises will be evaluated by the researchers, and the numerical value will be analyzed by a simple descriptive statistical method. We will perform a simple correlation analysis or simple regression analysis to determine whether there is a correlation between the differences in blood pressure and the intensity and difficulty of the exercise as perceived by the experimental group.

Information regarding adverse events will be collected through patient reports and researchers’ observations. The frequency of adverse events will be analyzed by a chi-squared test or Fisher’s exact test.

All statistical analyses will be conducted in a two-sided manner, with a significance level of 5%. In addition, we will use an intention-to-treat analysis for missing data in the primary and secondary analyses. The last-observation-carried-forward and multiple imputation, which are widely used in clinical research, will be applied to missing data and additional multiple imputation or regression analysis will be used to check any differences.

### Safety

Because the intervention is a simple exercise rather than untested drugs or medical devices, we do not expect any adverse events will be caused by the general Korean medical treatment. However, the investigators will inform the participants of all possible adverse events that may occur after taichi and instruct them to report any such adverse events. All adverse events recorded during the research period will be analyzed and reported. In general, due to the interventional characteristics of exercise, simple muscular pain may occur. We will provide beverages such as bottled water or green tea during the exercise sessions for the participants. To prevent falls and severe muscle aches, a chair will be available during exercise sessions. There will also be a space where participants can sit and relax. If a direct injury occurs in connection with this study, appropriate medical action may be taken, as determined by the investigator.

A data monitoring committee (DMC) comprising members of staff from the clinical research service institution (Woosuk University) will periodically monitor the study by telephone, e-mail, and visits, if necessary. The DMC will be composed of one statistician and one specialist in clinical research methodology. They will review the progress of the study and check all case report forms. In addition, they will check whether the study has followed the study protocol. If a problem is identified, the DMC will modify the protocol if necessary. If modifications to the protocol are required during the study, the modified protocol will be submitted by the clinical trial review committee to the IRB for approval. However, neither auditing nor an interim analysis are planned during the trial.

Additionally, as we will not collect any biological samples and do not intend to use the participants’ data in future studies, we do not need any additional consent for collection and use of participant data or biological specimens in ancillary studies. In accordance with government regulations and standards, all documents related to the conduct of a clinical trial must be kept by the director of clinical research, or the director of the institution and the clinical research manager, for at least 3 years after the completion of the clinical trial.

### Ethics and dissemination

As this clinical study was prepared with patient rights and well-being in mind based on the Declaration of Helsinki, the clinical researchers will follow the research plan and will actively respond to any problems raised by the participants. Additional file 1 contains a completed SPIRIT checklist.

Before patients are asked to participate in this clinical study, the researchers will explain to them all the details of the research. They must voluntarily agree to participate in the research. The English initials of the names of the participants in the trial will be recorded and identifying information will be managed using a subject identification code list to prevent personal information, such as social security numbers, from being leaked. Other researchers and research organizations will be able to view the clinical research data collected during reviews by the DMC, IRB inspections and assessments, and government surveys. On completion of the trial, the researchers will consult with the clinical research service institution (Woosuk University) to prepare a report of this study. The study findings will be disseminated in peer-reviewed journals and presented at national and international conferences. Additionally, we will share deidentified individual patient data. The datasets used or analyzed during the study can be requested from the corresponding author.

## Discussion

When searching for research conducted in Korea into the effects of taichi on blood pressure, we found studies that assessed the effects of taichi on waist circumference and blood pressure of the elderly [18] and the effect of taichi on the cardiac autonomous nervous system and blood pressure of elderly women [16]. These studies on taichi mainly had elderly participants. In addition, in a systematic review by Hwang et al. [19], only randomized controlled trials from China, Italy, United Kingdom, USA, Hong Kong, and Taiwan were analyzed, and no randomized controlled trials from Korea were included.

A major limitation of this study is that the participants cannot be blinded due to the nature of the intervention. Because the experimental group has to perform taichi, the participants will know whether they belong to the experimental group or the comparator group. This knowledge may affect the results of the study by influencing other behaviors, which may, thus, differ between the groups.

A strength of this study is that it will evaluate the effects of taichi in ordinary adults aged 19 to 70 years instead of being limited to the elderly. In addition, this study is a randomized controlled trial of taichi, which is not widely practiced in Korea. Taichi is easy to perform and not strenuous, so it can easily be practiced at home by almost anyone. As a result, this study may provide valuable data on the effects of taichi on hypertension.

### Trial status

The current protocol is version 1.7, dated 28 November 2019. Recruitment began on 17 April 2019. The approximate completion date for recruitment is in April 2020. This trial was prospectively registered before recruitment began.

## Supplementary information

**Additional file 1.** SPIRIT 2013 Checklist: Recommended items to address in a clinical trial protocol and related documents.

## Data Availability

The datasets used or analyzed during the study can be requested from the corresponding author.

## Abbreviations

CRC: Clinical research coordinator
DBP: Diastolic blood pressure
DMC: Data monitoring committee
IRB: Institutional review board
PNUKH: Pusan National University Korean Medicine Hospital
PNUYH: Pusan National University Yangsan Hospital
SBP: Systolic blood pressure
